# Cord blood DNA methylation modifications in infants are associated with white matter microstructure in the context of prenatal maternal depression and anxiety

**DOI:** 10.1038/s41598-021-91642-0

**Published:** 2021-06-09

**Authors:** Douglas C. Dean, Andy Madrid, Elizabeth M. Planalp, Jason F. Moody, Ligia A. Papale, Karla M. Knobel, Elizabeth K. Wood, Ryan M. McAdams, Christopher L. Coe, H. Hill Goldsmith, Richard J. Davidson, Reid S. Alisch, Pamela J. Kling

**Affiliations:** 1 Department of Pediatrics, School of Medicine & Public Health, University of Wisconsin-Madison, Madison, USA; 2 Department of Medical Physics, School of Medicine & Public Health, University of Wisconsin-Madison, Madison, WI USA; 3 Waisman Center, School of Medicine & Public Health, University of Wisconsin-Madison, Madison, WI USA; 4 Department of Neurosurgery, School of Medicine & Public Health, University of Wisconsin-Madison, Madison, WI USA; 5 Department of Psychology, School of Medicine & Public Health, University of Wisconsin-Madison, Madison, WI USA; 6 Harlow Center for Biological Psychology, School of Medicine & Public Health, University of Wisconsin-Madison, Madison, WI USA; 7 Center for Healthy Minds, School of Medicine & Public Health, University of Wisconsin-Madison, Madison, WI USA; 8 Department of Psychiatry, School of Medicine & Public Health, University of Wisconsin-Madison, Madison, WI USA

**Keywords:** Paediatric research, Development of the nervous system, Brain

## Abstract

Maternal and environmental factors influence brain networks and architecture via both physiological pathways and epigenetic modifications. In particular, prenatal maternal depression and anxiety symptoms appear to impact infant white matter (WM) microstructure, leading us to investigate whether epigenetic modifications (i.e., DNA methylation) contribute to these WM differences. To determine if infants of women with depression and anxiety symptoms exhibit epigenetic modifications linked to neurodevelopmental changes, 52 umbilical cord bloods (CBs) were profiled. We observed 219 differentially methylated genomic positions (DMPs; FDR *p* < 0.05) in CB that were associated with magnetic resonance imaging measures of WM microstructure at 1 month of age and in regions previously described to be related to maternal depression and anxiety symptoms. Genomic characterization of these associated DMPs revealed 143 unique genes with significant relationships to processes involved in neurodevelopment, GTPase activity, or the canonical Wnt signaling pathway. Separate regression models for female (n = 24) and male (n = 28) infants found 142 associated DMPs in females and 116 associated DMPs in males (nominal *p* value < 0.001, R > 0.5), which were annotated to 98 and 81 genes, respectively. Together, these findings suggest that umbilical CB DNA methylation levels at birth are associated with 1-month WM microstructure.

## Introduction

The first years of life are characterized by dynamic brain growth that is governed by genetic and environmental factors which help shape the structural and functional architecture of the brain and provide a foundation for subsequent cognition, behavior, and well-being^1,2^. In particular, recent neuroimaging studies indicate maternal depression and anxiety symptoms impact early brain development of the mother’s children, with higher depression and anxiety symptoms associated with reductions in cortical thickness and regional brain volumes^3–5^, differing patterns of functional connectivity^6,7^, and alterations in the maturing WM microstructure^5,8–14^. Despite evidence that maternal factors influence the brain prenatally, the molecular mechanisms underlying the effects on infant neurodevelopment associated with maternal depression and anxiety remain unclear.

Genome-wide DNA methylation levels vary dramatically throughout life and are responsive to early life adversity, including prenatal stress, separation from parents or variable maternal care^15–18^. DNA methylation (5-methylcytosine [5mC]) is an environmentally sensitive epigenetic modification serving important functions in chromatin remolding, gene silencing, embryonic development, cellular differentiation, and the maintenance of cellular identity^19^. Moreover, alterations in DNA methylation levels are emerging as important factors in the long-term biological trajectories leading to stress-related psychiatric disorders^20,21^. For instance, changes in 5mC levels in adults have been linked psychiatric disorders, including depression, anxiety, post-traumatic stress disorders and schizophrenia^22–25^. These studies suggest that DNA methylation is associated with neurobehavioral disorders in adults; however, the molecular mechanisms contributing to these disorders are likely to be initiated much earlier than adulthood^26–28^. In particular, placental and fetal DNA methylation levels provide tools to better understand the impact of prenatal depression and anxiety symptoms on molecular pathways in the infants. These indices may provide insights into the cellular and intracellular mediating pathways that contribute to the neurodevelopmental effects associated with prenatal anxiety and depression symptoms^29,30^.

Much remains to be learned about the role that adverse prenatal exposures have on DNA methylation levels and early brain development. A focused study on DNA methylation profiles from umbilical cord blood (CB) may potentially provide novel diagnostic tools to help identify at-risk infants and to improve assessment and clinical treatment strategies^27,31^. Previous work indicates prenatal exposure to maternal depression and anxiety symptoms can influence the developing brain, and in particular, the white matter (WM) microstructure^3–14,32,33^. In Dean et al. (2018), we observed associations between measures infant WM microstructure and prenatal maternal depression and anxiety symptoms^8^, including several WM regions, localized in regions spanning the corona radiata, superior longitudinal fasciculus, posterior thalamic radiations, among others, in which the association differed between male and female infants. To identify potential molecular mechanisms contributing to these observed sex-by-prenatal maternal depression and anxiety symptom interactions, here we examine the association between DNA methylation levels in cells from CB at delivery and measures of 1-month WM microstructure from regions identified in Dean et al. (2018). Specifically, we performed genome-wide DNA methylation profiling of infant leukocytes and examined how DNA methylation levels related to the WM microstructure in the preselected regions. Since the association between maternal depression and anxiety symptoms and 1-month WM microstructure was found to differ between males and females; we additionally performed sex-specific analyses. In sum, the aim was to identify genes and pathways involved in WM development that might be regulated by modifiable molecular substrates (e.g., DNA methylation), and that ultimately could be amenable to early intervention and treatment.

## Methods

### Study design and participants

Participants (*N* = 55 mother-infant dyads) were selected from a larger longitudinal study investigating the influence of early-life experience on child brain development^8,34–36^. Eligibility for the overall study included pregnant women aged 18–40 years, without major psychiatric illness (e.g., schizophrenia, bipolar or borderline personality disorder), or major autoimmune or infectious disorders during pregnancy. Infants were included if singleton, born between 37 and 42 weeks, and if discharged with the mother, without neurologic conditions, major birth head trauma, or a neonatal intensive care unit stay beyond observation^8,34–36^. Additional inclusion criteria for the current study included both an available CB sample collected at the time of delivery and a successful diffusion weighted MRI of the infant at 1 month of age. The University of Wisconsin–Madison Institutional Review Board approved all study procedures. Written informed consent was obtained from all mothers and written informed parental consent was obtained for all infants. All experiments were performed in accordance with relevant guidelines and regulations.

### Prenatal maternal depression and anxiety symptoms

Mothers completed the Edinburgh Postnatal Depression Scale (EPDS)^37^ and State-Trait Anxiety Inventory (STAI)^38^ at 28 and 35 weeks of gestation. The EPDS is a widely used 10-item screening tool for perinatal depression, with scores ranging from 0 to 30 and a cut-off value of 10 or higher typically used to identify women who endorse depressive symptoms over the last month^39^. The 20-item STAI measures both state (14-items) and trait (6-items) level anxiety. Here, we focused on state anxiety, which captures maternal anxiety symptoms over the last month, with scores ranging from 0 to 42. As previously described, EPDS and STAI scores were strongly correlated, and a principal component analysis was used to construct a psychometrically valid depression and anxiety composite score from the prenatal EPDS and STAI measures^8^. This composite score was positively correlated with 28- and 35-week EPDS and STAI scores, with higher scores indicating more maternal symptoms during pregnancy. This composite score was used in subsequent analyses.

### Cord blood collection and DNA extraction

After delivery, umbilical CB for whole blood genomic DNA was collected in sodium heparin vacutainer tubes. Whole cord blood samples were stored at 4 °C and processed at a median of 7.85 h after delivery (range 1.75–135 h, with two-thirds processed within 12 h and all but one before 48 h). gDNA was isolated by QIAamp DNA Mini Kit spin column protocol (Qiagen), quantitated by PicoGreen DNA dye (Turner BioSystems), using human gDNA standards (Promega E3401). DNA samples were stored frozen at − 70 °C.

### Diffusion imaging data acquisition and processing

MRI was performed on a 3T scanner (MR750 Discovery scanner; General Electric) with a 32-channel head RF array coil (Nova Medical). Scanning was performed when the infant was 1-month of age during natural, nonsedated sleep^34,35,40^. Measures taken for successful image acquisition included acoustic noise reduction via fitting a foam insert inside the MRI scanner bore, utilizing ear plugs and MiniMuff (Natus Medical Incorporated) neonatal noise-attenuating ear covers, playing white noise through electrodynamic headphones (MR Confon, Germany) throughout the image acquisition, and limiting the MRI scanner slew rates. To help reduce motion throughout the scan, we swaddled each child using an infant MedVac vacuum immobilization bag and placed foam cushions around the head. Additional details about MRI acquisition have been previously described^8,34–36,40^.

A 10-min multi-shell diffusion imaging protocol was used to acquire 69 diffusion weighted images (DWIs), with 9/18/36 diffusion-encoding gradient directions at b = 350/800/1500 s/mm^2^, respectively, and 6 with no (b = 0 s/mm^2^) diffusion weighting. Additional imaging criteria consisted of the following: image resolution 2 × 2 × 2 mm; repetition time, 8400 ms; and echo time, 94 ms. DWI data were manually inspected for image artifacts, eddy current and motion corrected^41^, and skull-stripped (http://afni.nimh.nih.gov/pub/dist/doc/program_help/3dSkullStrip.html). Diffusion tensor imaging (DTI^42^) and Neurite Orientation Dispersion and Density (NODDI^43^) parameters were estimated and registered to a common, population specific template. Additional details regarding the diffusion image acquisition and processing are described elsewhere^8,34^.

**Figure 1:**
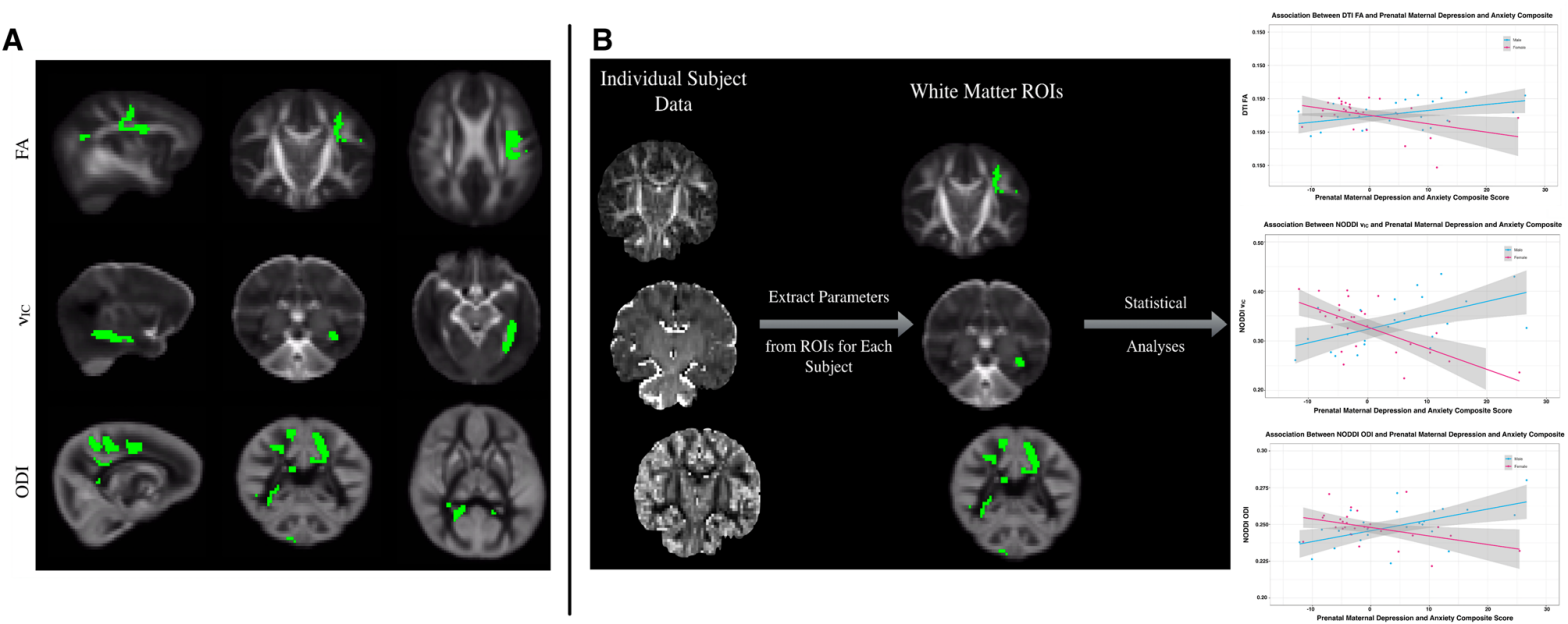
(**A**) Representative sagittal, coronal, and axial sections of WM regions previously found to be differentially associated with maternal depression and anxiety symptoms during mid-late pregnancy. DTI FA associations were observed in regions of the corona radiata and superior longitudinal fasciculus, among others; NODDI ν_IC_ associations were found in the sagittal stratum and white matter adjacent to the hippocampus; and NODDI ODI associations were observed in areas of posterior thalamic radiations, splenium of the corpus callosum, among others^8^. (**B**) Schematic illustrating extraction of WM microstructural measures from WM regions. Mean FA, ν_IC_, and ODI were extracted from the respective WM regions for each subject and used in subsequent analyses.

Three WM regions previously identified to have differing associations between prenatal maternal depression and anxiety symptoms and DTI fractional anisotropy (FA), NODDI intracellular volume fraction (ν_IC_), and NODDI orientation dispersion index (ODI) in males and females were examined. These regions spanned different areas of WM (Fig. 1A), including regions of the corona radiata and superior longitudinal fasciculus, among others (FA); the sagittal stratum and white matter adjacent to the hippocampus (ν_IC_); and regions of posterior thalamic radiations, splenium of the corpus callosum, among others (ODI)^8^. For each subject, mean FA, ν_IC_, and ODI values were extracted from the corresponding WM regions and used in subsequent DNA methylation analyses. Figure 1B provides a schematic of how average WM microstructures measures were computed for each subject.

### Preprocessing of human methylation EPIC data

Genome-wide DNA methylation levels were determined using the Infinium HumanMethylationEPIC array (Illumina, San Diego, CA) and raw intensity data files were imported into the R environment^44^. The R package *minfi* was used to assess sample quality, calculate the signal detection *p* value of each tested probe, estimate cell proportions in the CB specimens, predict sex, and normalize data^45,46^. Two samples were discarded because they did not pass quality control, as their mean signal detection *p* value exceeded 0.05. Probes were normal-exponential out-of-band (noob) normalized with dye correction, followed by quantile normalization. One additional sample was discarded as it failed sex prediction, suggesting unreliable DNA methylation levels for this sample. Probes were filtered if any one sample exhibited a signal detection *p* value > 0.01, contained a SNP, reported methylation at a SNP, were derived from sex chromosomes, measured methylation at a CH dinucleotide site, or are known cross-reactive probes (≥ 47 bp homology with an off-target site)^47,48^. These filtration criteria resulted in 763,917 probes used for further analysis.

### Identification of differentially methylated loci

Beta-values were obtained through *minfi* and were further converted to logit-transformed *M*-values for differential analyses. Linear regression for each tested CpG using a multivariate model was employed using the R package *limma*^49^. For regression, separate models for key WM microstructure measures of FA, ν_IC_, or ODI were constructed, controlling for CB cell proportions (i.e., CD8+T, CD4+T, Natural Killer, Monocyte, B cell, and Granulocyte), sex, infant gestational age-corrected postpartum age, Hollingshead socioeconomic status^50^, and an index of total motion during the diffusion acquisition^51^; gene length was not considered in the analysis. Surrogate variables were assessed by the R package *sva*^52^ and identified 6 surrogate variables that were adjusted for during model fitting*.* To assess systematic bias of the linear regression model, the genomic inflation factor was calculated for the obtained *p* values, yielding a genomic inflation factor of ~ 1, suggesting no bias. Pearson’s correlation coefficients (r) were calculated for continuous variables of interest with beta-values. Differential methylation was deemed significant if the Benjamini–Hochberg FDR adjusted *p* value < 0.05 (full data) or the unadjusted *p* value < 0.001 and the correlation (r) was > 0.5 (sex-specific data). Notably, minimizing family-wise error rates with conventional false discovery rates is overly conservative for methylation data, because DNA methylation levels are continuous variables when measured across a large number of cells and neighboring probes on the array are known to be correlated and many sites on the array are non-variable^53,54^. Thus, the DMPs identified in the full dataset are likely a conservative set. The smaller sample size of the sex-specific analysis likely resulted in the absence of FDR adjusted DMPs.

### Statistical analyses

The distributions of demographic data were evaluated. Comparisons between female and male infants were conducted with *t* tests for continuous and χ^2^ for categorical variables, with *p* value < 0.05 considered significant. Infants were included if mothers’ prenatal depression and anxiety scores were obtained, CB volumes were sufficient for obtaining DNA for epigenetic analysis, and full NODDI MRI imaging data were obtained.

Genes exhibiting differential methylation were subjected to gene ontological analysis using the R package *clusterProfiler*^4^ to identify significant disruptions in biological processes, and to define gene concepts and map enrichment terms. The database used for the gene pathway analysis was the “Genome wide annotation for Human” (org.Hs.eg.db) in the following Bioconductor R package: https://bioconductor.org/packages/release/data/annotation/html/org.Hs.eg.db.html. The total number of genes subjected to the differential analysis was used as the background gene universe. Pathways were included in the analysis if they had a at least 10 and no more than 500 genes in the background gene set. Gene ontological terms were deemed significant if *p* value < 0.05.

## Results

### Cohort demographics

From a total 149 possible mother-infant dyads, 73 had insufficient CB volumes for DNA extraction or did not pass quality control procedures (see “Methods” section), and an additional 24 infants did not complete the entire MRI scan, resulting in a final sample of 52 (24 female). Demographic data are shown in Table 1. Infants were healthy at birth, without differences in demographic characteristics between females and males, except that more males were born in middle-income families and thus fewer males were born into the high-income families. Mothers with male infants reported higher state trait anxiety index (STAI) at 35 weeks of gestation (*p value* = 0.04) and tended to have a higher depression and anxiety composite score (*p* value = 0.07). Twenty-four mothers (46.2%) reported EPDS scores between 0 and 6, 17 mothers (32.7%) reported EPDS scores between 7 and 10, and 11 mothers (21.2%) reported EPDS scores ≥ 11. Six mothers reported EPDS scores higher than 12, which is generally consistent with a diagnosis of major depressive disorder. Six women reported antidepressant medication use for depression or anxiety during pregnancy. The EPDS scores and STAI scores conveyed relatively mild to moderate levels of depression and/or anxiety symptomology.

**Table 1:** Demographic information for study cohort.

	Combined	Females	Males	*p* value
**Infant characteristics**				
N	52	28	24	
Gestation length (weeks)	39.81 (1.16)	39.79 (1.13)	39.84 (1.23)	0.88
Birth weight (kg)	3.59 (0.51)	3.54 (0.52)	3.64 (0.51)	0.5
Birth length (cm)	52.07 (2.58)	51.77 (2.58)	52.46 (2.59)	0.36
Birth head circumference (cm)	34.81 (1.38)	34.45 (1.21)	35.29 (1.47)	0.05
5 min APGAR score	8.92 (0.34)	8.89 (0.42)	8.95 (0.21)	0.48
Gestation corrected age at MRI (days)	32.79 [19–50]	32.46 [19–50]	33.17 [22–42]	0.69
**Maternal characteristics**				
Age at birth (years)	32.87 (4.13)	33.33 (3.69)	32.32 (4.60)	0.39
Years of education	32.87 (4.14)	17.50 (2.57)	17.58 (2.57)	0.91
Family income				
$30,001–50,000	7 (13.5%)	4 (14.3%)	3 (12.5%)	0.04
$50,001–100,000	22 (42.3%)	7 (25.0%)	15 (62.5%)	
$100,001–200,000	22 (42.3%)	16 (57.1%)	6 (25.0%)	
Not reported/missing	1 (1.9%)	1 (3.6%)	0 (0%)	
Marital status				
Married	46 (88.5%)	24 (85.7%)	22 (91.7%)	0.52
Single	2 (3.8%)	1 (3.6%)	1 (4.2%)	
Divorced/separated	2 (3.8%)	1 (3.6%)	2 (8.3)%	
Not reported/missing	2 (3.8%)	2 (7.1%)	0 (0%)	
Race				
African American/Black	2 (3.8%)	2 (7.1%)	0 (0%)	0.33
Asian	2 (3.8%)	1 (3.6%)	1 (4.2%)	
Caucasian/White	44 (84.6%)	22 (78.6%)	22 (91.7%)	
Native Hawaiian or other Pacific Islander	2 (3.8%)	2 (7.1%)	0 (0%)	
Mixed race	1 (1.9%)	1 (3.6%)	0 (0%)	
Not reported/missing	1 (1.9%)	0 (0%)	1 (4.2%)	
Medication status				
Antidepressants	6 (11.5%)	1 (3.6%)	5 (20.8%)	0.13
Corticosteroids	2 (3.8%)	2 (7.1%)	0 (0%)	
Hormones	2 (3.8%)	2 (7.1%)	0 (0%)	
Pain relief or nonsteroidal	13 (25.0%)	6 (21.4%)	7 (29.2%)	
None	32 (61.5%)	18 (64.3%)	14 (58.3%)	
Alcohol use during pregnancy	19 (36.5%)	10 (35.7%)	9 (37.5%)	0.9
Tobacco use during pregnancy	1 (1.9%)	0 (0%)	1 (4.2%)	0.28
Delivery method				
Vaginal	37 (71.2%)	20 (71.4%)	17 (70.8%)	0.12
Cesarean section	12 (23.1%)	8 (28.6%)	4 (16.7%)	
Not reported/missing	3 (5.8%)	0 (0%)	3 (12.5%)	
Edinburgh Postnatal Depression Scale				
28 week	7.24 (4.74)	6.42 (4.25)	8.13 (5.17)	0.21
35 week	6.34 (4.07)	5.36 (3.78)	7.50 (4.17)	0.06
State Trait Anxiety Index				
28 Week	11.62 (6.95)	10.54 (6.67)	12.79 (7.20)	0.26
35 Week	12.25 (5.69)	10.71 (4.58)	14.04 (6.40)	0.04
Depression/anxiety composite score	1.99 (9.28)	-0.23 (7.98)	4.57 (10.15)	0.07
Hours between birth and cord blood processing	15.6 (24.0)	21.7 (33.0)	9.8 (7.5)	0.16

Data are presented as mean (standard deviation) or number (percentage), as indicated. *P* values correspond to comparisons between males and females using T-tests or Chi-squared tests, where appropriate.

### Sex-differing associations between prenatal maternal depression and anxiety symptoms and infant WM microstructure

To determine the extent to which CB DNA methylation levels were correlated with sex-differentiated relationships with prenatal maternal depression and anxiety symptoms, genomic DNA was analyzed from CB using the HumanMethylationEPIC beadchip array (Illumina). After filtering out unreliable probes (see “Methods” section), this analysis provided a reliable quantitative measure of DNA methylation levels at 763,917 CpG dinucleotides across the human genome at single-nucleotide resolution, including enhancers and all coding regions. A regression model using late pregnancy depression and anxiety composite scores during late pregnancy as the explanatory variable did not find correlations with CB DNA methylation levels after FDR correction; however, 130 DMPs corresponding to 91 genes were correlated at a lower stringency (*p* value < 0.001, R > 0.4).

In Dean et al. (2018), associations between prenatal maternal depression and anxiety symptoms and infant WM microstructure were previously observed to differ between males and females in the larger sample (*N* = 101)^8^. CB samples were available from only a subset of these infants (*N* = 52). Therefore, to confirm the previous findings, linear regressions using mean FA, ν_IC,_ and ODI measures were repeated for the smaller subset with an available CB specimen. The sex-by-prenatal maternal depression and anxiety symptom interactions remained significant for FA, ν_IC_ and ODI in the smaller sample (*p* value = 0.002, < 0.001, < 0.001, respectively; Supplementary Fig. 1), suggesting lower FA, ν_IC_ and ODI in females and higher FA, ν_IC_ and ODI in males exposed to higher levels of maternal depression and anxiety. These WM measures at 1 month of age were used to determine if there were significant correlations with CB DNA methylation levels.

### Differential methylation related to infant WM microstructure

**Figure 2:**
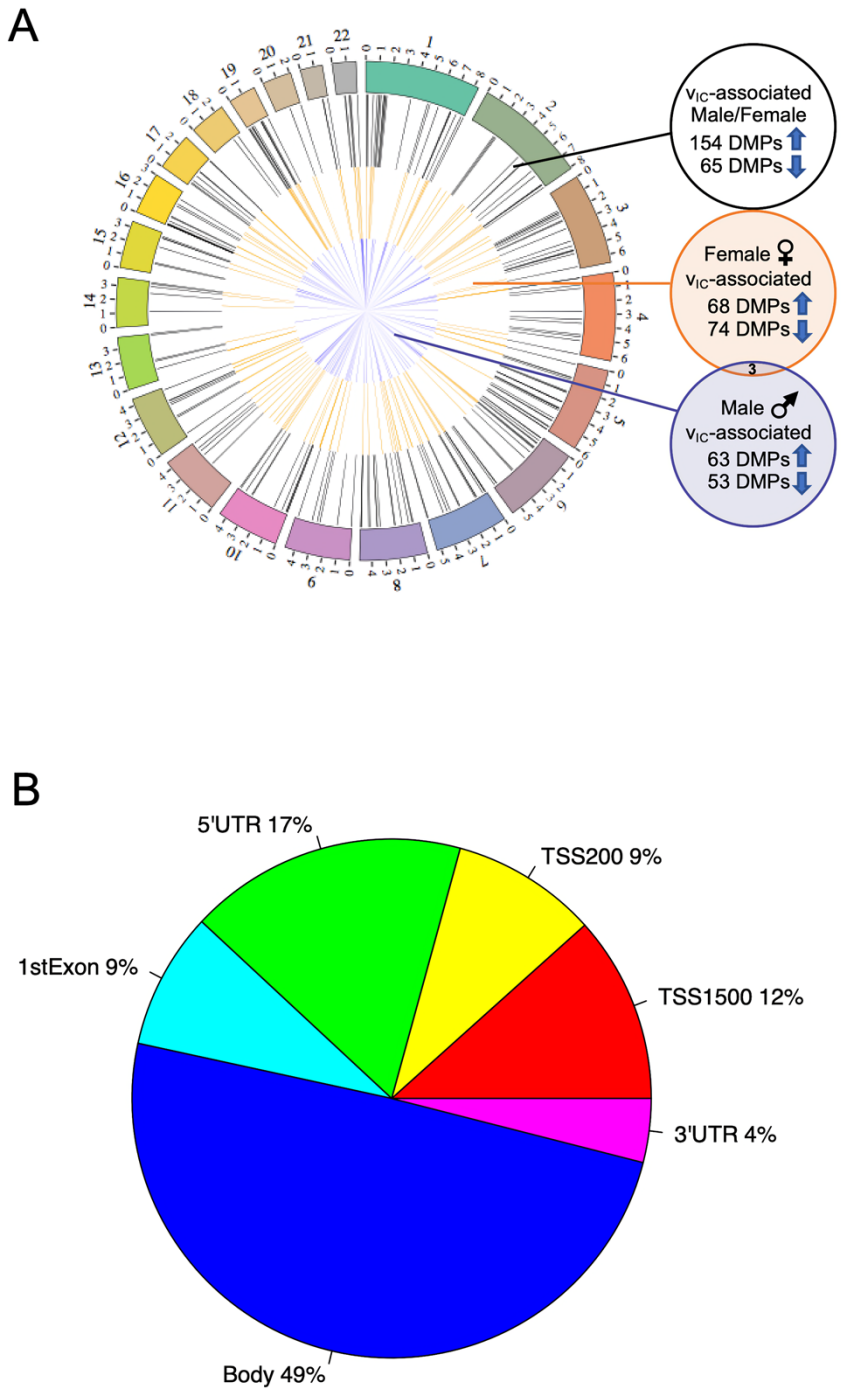
Cord blood DNA methylation levels associated with infant WM microstructure-related changes (ν_IC_) showing a sex-by-prenatal maternal depression and anxiety symptom interaction. (**A**) Circos plot depicts genomic distribution of differentially methylated positions (DMPs) across the human genome. (Outer ring) Each chromosome is shown as a different color and the relative chromosome size is represented by the bar length. (Inner rings) Represent the relative location of DMPs across all chromosomes in the full dataset (black), females only (orange), and males only (blue). Sex chromosomes were omitted from the analysis. B) Pie chart showing the percent distribution of DMPs to standard genomic features. 5′UTR = 5′ untranslated region’ 3′UTR = 3′ untranslated region; TSS = transcription start site; TSS200 = 0–200 bp upstream of TSS; TSS1500 = 200–1500 bp upstream of TSS. Circos plot and pie chart were generated using the R environment^44^.

Individual regression models for 1-month infant FA, ν_IC_, and ODI at 1 month were performed, with the WM microstructure indices considered as the explanatory variable. No correlation between DNA methylation levels in CB and FA and ODI that survived FDR correction; however, 319 (FA) and 122 (ODI) DMPs corresponding to 217 (FA) and 99 (ODI) genes were found at lower a stringency (*p* value < 0.001, R > 0.4). In contrast, we found 219 ν_IC_-associated DMPs in CB (FDR *p* value < 0.05; Fig. 2A; Dataset 1). These ν_IC_-associated DMPs were distributed across all autosomes with a total of 154 hyper- and 65 hypo-DMPs (Dataset 1), indicating the majority of sites contributing to WM microstructure had higher DNA methylation levels.

Annotation of the ν_IC_-associated DMPs to standard genomic structures revealed that nearly half (47%) resided in promoter regions of annotated genes (i.e., within 1,500 base pairs of the transcription start site or the first exon; Fig. 2B). Annotation of the 219 ν_IC_-associated DMPs to genes revealed 143 unique genes, including genes known to contribute to the development of the nervous system (*e.g., LDLR* and *KDM4A*).

### Differential methylation regions are linked to nervous system and Wnt signaling pathways

**Figure 3:**
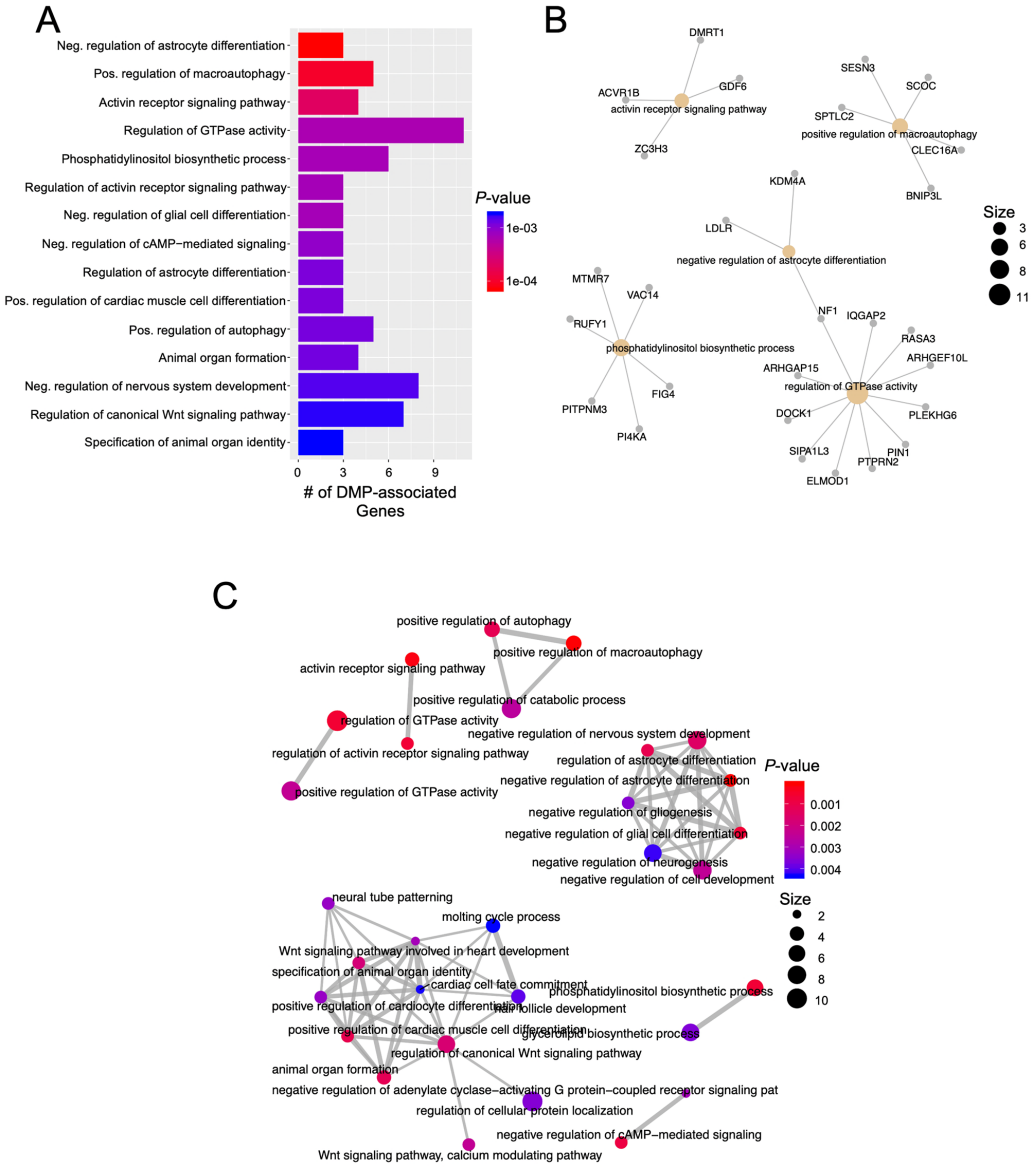
Gene pathways analysis of differentially methylation position (DMP) in CB that were associated infant WM microstructure-related changes (ν_IC_) showing a Sex x Symptom interaction. (**A**) Bar Graph of the top 20 gene ontological (GO) biological processes associated with the differentially methylated genes, ordered by statistical significance. X-axis indicates the number of DMP-associated genes contributing to each GO term. The shade of the bars shows the *p* value based on the legend, as determined by a Fischer test. Neg. = Negative; Pos. = Positive. (**B**) Gene-concept network plot shows the top five gene ontology terms (beige), the genes (gray) associated with each term, and the interconnectivity between genes and processes (lines). The size of the beige dot relates to the number of DMP-associated genes contributing to that term. (**C**) An enrichment map plot depicts the connectivity of associated terms, with hubs of similar processes clustering further apart. Node (spheres) size represent the relative number of DMP-associated genes contributing to each term, while the color represents the FDR *p*-value, shown in the legend, as determined by a Fischer test. The size of the edges (gray lines) depicts the strength of relatedness between terms. Bar graph, gene-concept network plot, and enrichment map were constructed using the R environment^44^.

To characterize genes and pathways linked to infant WM microstructure at 1 month of age, we next examined the gene ontologies (GO) of the ν_IC_-associated genes that had DMPs. These analyses revealed significant relationships between genes linked to processes involved in astrocyte differentiation, GTPase activity, and Wnt signaling (Fig. 3A,B; Supplementary Table 1). Moreover, gene network mapping of the enrichment results identified two major gene network hubs: nervous system development, including negative regulators of nervous system development, and regulation of the canonical Wnt signaling pathway (Fig. 3C).

### Sex-specific differential methylation related to infant WM microstructure

Associations between ν_IC_ and prenatal maternal depression and anxiety symptoms that differed by sex^8^ led us to investigate sex-specific DNA methylation levels in relation to infant WM microstructure at 1 month. Using separate regression models for females (*N* = 24) and males (*N* = 28), we observed 142 ν_IC_-associated DMPs in females and 116 ν_IC_-associated DMPs in males (*p* value < 0.001, R > 0.5; Fig. 2A). These ν_IC_-associated DMPs were annotated to 98 and 81 genes in females and males, respectively, with an overlap of 3 genes (Chondroitin Sulfate *N*-Acetylgalactosaminyltransferase 1 [*CSGALNACT1*], Phosphodiesterase 6B [*PDE6B*], and Transcription Factor 7 Like 2 [*TCF7L2*]; Dataset 1). There was only one female-specific DMP-associated gene (*ACVR1B*) and one male-specific DMP-associated gene (*TLL2*) that overlapped with the full dataset.

## Discussion

This study extends our prior findings linking WM microstructure at 1 month of age to maternal depression and anxiety symptoms during the prenatal period^8^ and now identifies some associations with epigenetic modifications. Using CB leukocytes collected at delivery, we show that DNA methylation levels associated with the microstructure of several selected WM regions were enriched in gene pathways that negatively regulate neurodevelopmental processes.

Previous research examining the effect of maternal depression and anxiety on CB DNA methylation levels has yielded mixed results. A meta-analysis of two large birth cohorts with CB showed no epigenome-wide associations with prenatal stress^55^. Another study found 23 DMPs associated with depression in late pregnancy^30^. Although we did not find a robust, simple relationship between DMPs and prenatal depression and anxiety scores, we did find many DMPs associated with WM microstructure that were related to maternal depression and anxiety during the third trimester. These findings support the value of employing a targeted approach utilizing microstructural brain imaging to identify anatomical regions associated with CB DNA methylation levels.

DMPs were associated with WM neurite density in the left-hemisphere sagittal stratum^8^, a major WM bundle containing the inferior fronto-occipital fasciculus, inferior longitudinal fasciculus, and posterior thalamic radiations^56–58^. Several underlying processes contribute to the development of these WM tracts, including astrocyte, glial, neuronal and cellular proliferation and differentiation^59^. ν_IC_-associated DMPs were detected on genes that negatively regulate astrocyte differentiation, including the low-density lipoprotein R (*LDLR)*, Neurofibromin 1 (*NF1*), Wingless-Type MMTV Integration Site Family, Member 3A (*WNT3A*) and patched-1 (*PTCH1*). *LDLR* functions as a carrier of cholesterol in blood, which is important in neuronal differentiation and synaptogenesis^60^. *NF1* encodes a protein that regulates cell growth through the Ras pathway, with *NF1* dysregulation affecting brain development^61–63^, including WM microstructure^64,65^ and myelin^66^. *NF1* is also linked to the regulation of the GTPase pathway, which plays a role in ectoderm differentiation and is linked to microcephaly, structural brain abnormalities, and intellectual disability^67–69^. Finally, *WNT3A* and *PTCH1* provide proteins that trigger signals essential for cell fate, specialization, and patterning during embryogenesis^70,71^. These data suggest that ν_IC_-associated DNA methylation levels may play a role in the negative regulation of neuronal differentiation, growth, and patterning of WM microstructure.

We found unique sets of ν_IC_-associated alterations in DNA methylation levels for male and female infants, with an overlap of only three genes. Although the mechanisms underlying this difference are unknown, sex differences in DNA methylation levels may be related to variation in the timing of WM development between females and males, with WM usually maturing earlier in females than in males^72–74^. Moreover, environmental exposures during different gestational periods have unique effects on epigenetic programming of the developing embryo/fetus and influence males and females differently^75^. For example, stress-induced epigenetic changes during early gestation predominately affect male development in rats^76^, while stress during late gestation tends to have larger effects on development in females^76^. Thus, sex-specific effects may depend on the gestational timing of the adverse event. Whether these sex differences in DNA methylation are related to long-term neurodevelopmental and behavioral outcomes requires further study.

A descriptive analysis of the CB methylation events related to gene structure revealed that more than 50% of the ν_IC_-associated DMPs were located in the body of the gene, of which nearly 90% (70/79) were ν_IC_-associated decreases in DNA methylation levels. Because active transcription is generally associated with increased gene body DNA methylation levels^77^, the findings suggest that these 70 DMP-associated genes moderate gene expression in CB. Future studies are needed to confirm the function and expression levels of these candidate genes.

A strength of this study is the multi-method birth cohort design, which combined rich phenotypic data, CB samples collected at delivery for whole genome epigenetic analysis, and quantitative diffusion MRI for characterizing early brain development. Blood has the highest proportion of CpGs that are nominally correlated to brain, when compared to other available tissues, including buccal swabs and saliva^78^. Although the need for data across three domains limited our sample size, it allowed us to detect a novel association between the prenatal environment, CB molecular indices, and WM microstructure at 1 month of age. Importantly, the cord blood DNA methylation levels presented here could conceivably provide bioindicators that reflect how the prenatal environment shapes early brain development, independent of DNA methylation levels in brain. Another strength is the finding of DMPs on genes relevant to neurodevelopment in circulating nucleated blood cells; however, these DNA methylation changes may not be functional (i.e., alter gene expression) and thus still require further analysis. Further, despite the neuroimaging being performed in close proximity to birth, the influence of postnatal parenting during the first month of life could contribute to the findings. Our data are from a short developmental period; longitudinal studies are now needed to examine the impact of DNA methylation on subsequent neurodevelopmental trajectories at older ages.

Here, we identify a potential mechanism by which prenatal maternal depression and anxiety symptoms could negatively regulate the development of WM microstructure through epigenetic alterations. Although we had a relatively small cohort, several other studies have also found significant associations with processes or pathways in smaller cohorts^79–82^. The majority of mothers reported depression and anxiety scores in the subclinical range, limiting our ability to draw inferences about effects of overt maternal psychopathology, but presumably the alterations might be larger in clinically depressed mothers. In addition, only a few mothers reported using antidepressants during pregnancy, a potential limiting confounder, precluding our ability to examine the influence of medication use on DNA methylation and early brain development. Of note, previous analyses indicated no differences with the exclusion of these women^8^; however, future work would benefit from examining prenatal medication use in larger, longitudinal samples. Additional environmental or experiential factors not taken into account in the current study may also have some contributing impact on the processes investigated and should be considered in future studies of larger cohorts. Though not investigated here, the maturation of cortical and subcortical gray matter and functional connectivity may also be influenced by epigenetic modifications and are of interest for future study. Nonetheless, our findings provide evidence for an association between epigenetic modifications and WM microstructure even after the mild symptoms commonly experienced by a significant number of pregnant women^83^. Our findings suggest that these environmentally sensitive epigenetic modifications detected in umbilical CB are associated with altered WM microstructure at 1 month of age. The sex-specific differences in DNA methylation levels were present on genes that negatively regulate neurodevelopment, regulate GTPase, and modulate canonical Wnt signaling, and thus implicate candidate genes for further investigation of sex-related differences in risk for neurodevelopmental disorders. Understanding these early microstructural developmental patterns are essential for appreciating the changes that occur at later stages of growth and informs a deeper understanding of the role of epigenetic modifications on the development of early white matter and brain development.

## Supplementary information

Supplementary Information 1.

Supplementary Information 2.
