# Supplementary material for: Cord blood DNA methylation modifications in infants are associated with white matter microstructure in the context of prenatal maternal depression and anxiety
Source: Sci Rep. 2021 Jun 9;11:12181. doi: 10.1038/s41598-021-91642-0 (PMC8190282; doi:10.1038/s41598-021-91642-0)

**Cord Blood DNA Methylation Modifications in Infants are Associated with White Matter Microstructure in the Context of Prenatal Maternal Depression and Anxiety**

Douglas C. Dean III^1,2,3^, Andy Madrid^4^, Elizabeth M. Planalp^3,5^, Jason F. Moody^2^, Ligia A. Papale^4^, Karla M. Knobel^3^, Elizabeth K. Wood^6^, Ryan M. McAdams^1^, Christopher L. Coe^3,5,6^, H. Hill Goldsmith^3,5^, Richard J. Davidson^3,5,7,8^, Reid S. Alisch^4,*^, and Pamela J. Kling^1^

Departments of ^1^Pediatrics, ^2^Medical Physics, ^3^Waisman Center, ^4^Neurosurgery, ^5^Psychology, ^6^Harlow Center for Biological Psychology, ^7^Center for Healthy Minds, ^8^Psychiatry, University of Wisconsin-Madison, School of Medicine & Public Health.

*^*^Address Correspondence to:* Reid S. Alisch

University of Wisconsin-Madison

Madison, WI, USA, 53705

Tel. # +1 (608) 263-3793

Email: [alisch@wisc.edu](mailto:alisch@wisc.edu)

***Running Title: DNA Methylation Associates with Infant White Matter Microstructure***

Manuscript prepared for submission to Scientific Reports (Article)

Number of Supplemental Tables: 1

Number of Supplemental Figures: 1

**Supplementary Table 1:** The top 30 gene ontological (GO) terms.

**Supplementary Figure 1:** Linear regressions using mean FA, ν_IC,_ and ODI measures were repeated for the smaller subset with an available CB specimen. Previous sex-by-symptom interactions remained significant for FA, ν_IC_ and ODI (*p-*value = 0.002, < 0.001, < 0.001, respectively) for the smaller cohort. These WM measures at one month of age were used to determine if there were significant correlations with CB DNA methylation levels.


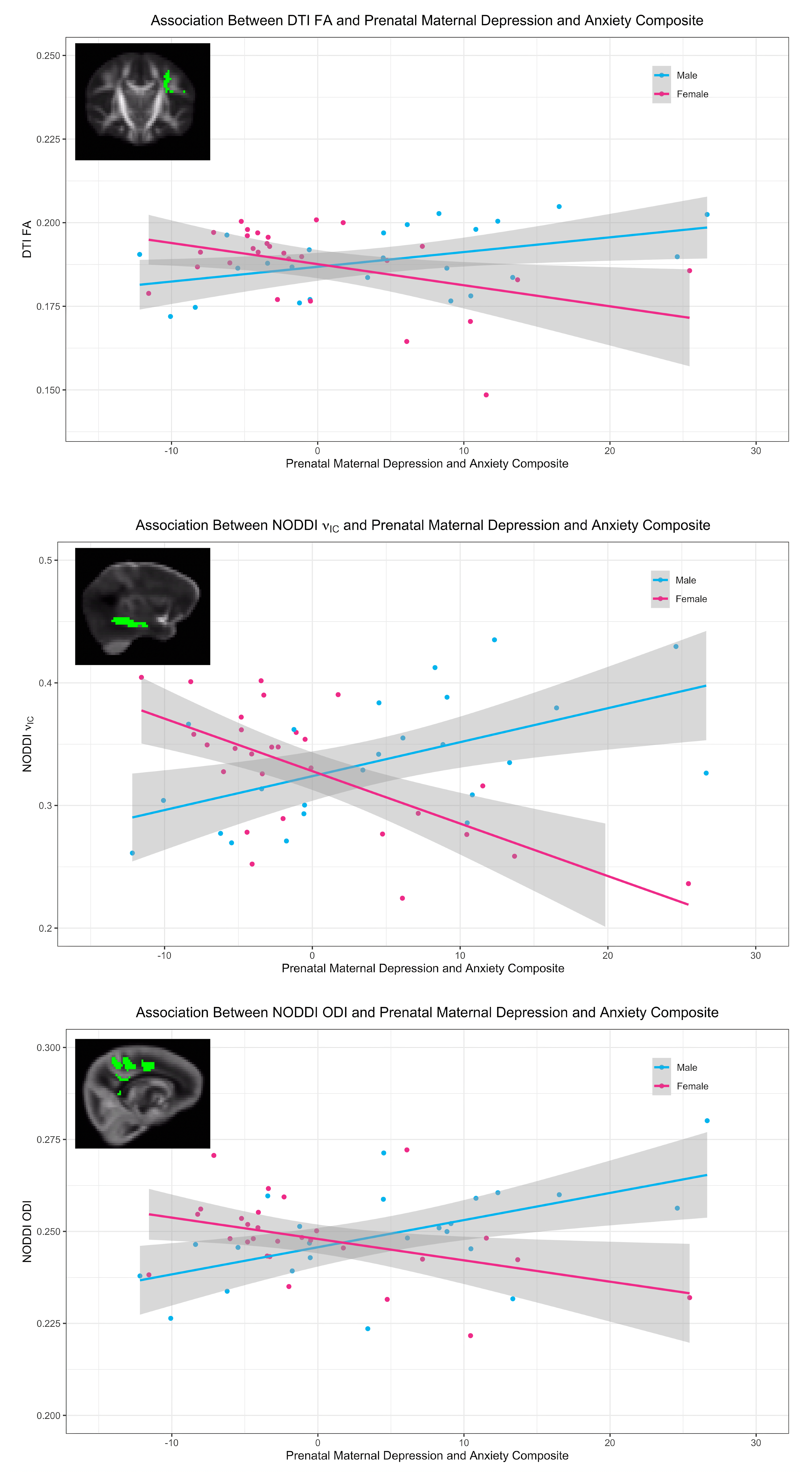

Supplement: Supplementary file 1 — Supplementary Information 1. [file 41598_2021_91642_MOESM1_ESM.docx]
